# Setting up a platform for plant-based influenza virus vaccine production in South Africa

**DOI:** 10.1186/1472-6750-12-14

**Published:** 2012-04-26

**Authors:** Elizabeth Mortimer, James M Maclean, Sandiswa Mbewana, Amelia Buys, Anna-Lise Williamson, Inga I Hitzeroth, Edward P Rybicki

**Affiliations:** 1 Department of Molecular and Cell Biology, University of Cape Town, Cape Town, South Africa; 2 Institute of Infectious Disease and Molecular Medicine, Faculty of Health Science, University of Cape Town, Cape Town, South Africa; 3 National Institute for Communicable Diseases, Modderfontein Road, Johannesburg, South Africa; 4 National Health Laboratory Service, Groote Schuur Hospital, Observatory, Cape Town, South Africa

## Abstract

**Background:**

During a global influenza pandemic, the vaccine requirements of developing countries can surpass their supply capabilities, if these exist at all, compelling them to rely on developed countries for stocks that may not be available in time. There is thus a need for developing countries in general to produce their own pandemic and possibly seasonal influenza vaccines. Here we describe the development of a plant-based platform for producing influenza vaccines locally, in South Africa. Plant-produced influenza vaccine candidates are quicker to develop and potentially cheaper than egg-produced influenza vaccines, and their production can be rapidly upscaled. In this study, we investigated the feasibility of producing a vaccine to the highly pathogenic avian influenza A subtype H5N1 virus, the most generally virulent influenza virus identified to date. Two variants of the haemagglutinin (HA) surface glycoprotein gene were synthesised for optimum expression in plants: these were the full-length HA gene (H5) and a truncated form lacking the transmembrane domain (H5tr). The genes were cloned into a panel of *Agrobacterium tumefaciens* binary plant expression vectors in order to test HA accumulation in different cell compartments. The constructs were transiently expressed in tobacco by means of agroinfiltration. Stable transgenic tobacco plants were also generated to provide seed for stable storage of the material as a pre-pandemic strategy.

**Results:**

For both transient and transgenic expression systems the highest accumulation of full-length H5 protein occurred in the apoplastic spaces, while the highest accumulation of H5tr was in the endoplasmic reticulum. The H5 proteins were produced at relatively high concentrations in both systems. Following partial purification, haemagglutination and haemagglutination inhibition tests indicated that the conformation of the plant-produced HA variants was correct and the proteins were functional. The immunisation of chickens and mice with the candidate vaccines elicited HA-specific antibody responses.

**Conclusions:**

We managed, after synthesis of two versions of a single gene, to produce by transient and transgenic expression in plants, two variants of a highly pathogenic avian influenza virus HA protein which could have vaccine potential. This is a proof of principle of the potential of plant-produced influenza vaccines as a feasible pandemic response strategy for South Africa and other developing countries.

## Background

During 2009, South Africa was faced with the novel (“swine”) influenza A H1N1 virus pandemic. As the country does not have the ability to produce influenza vaccine stocks, we had to rely on the World Health Organisation (WHO) and developed countries – and as expected, even developed countries did not have adequate vaccine stocks to meet their own demands. Luckily, the 2009 H1N1 pandemic virus caused only mild flu-like symptoms in most individuals. Certain groups of people, however, were at greater risk for serious disease complications, such children and young adults, people with diabetes, pregnant women and immunocompromised individuals [1]. Since South Africa carries a high disease burden which includes one of the highest Human immunodeficiency virus (HIV) prevalences in the world [2], this pandemic was a serious warning for South Africa to have a contingency plan in place for when the next influenza pandemic strikes. Ideally, South Africa should be self-sufficient; producing its own influenza vaccines to reduce morbidity and mortality in its large and relatively poor population.

Even routine seasonal influenza vaccine production by the traditional egg-based technology is expensive and slow, taking up to 6 months to complete from the notification by WHO of suitable seasonal strains [3]. This is not ideal for a pandemic situation, or for a developing country with limited funds available. At a WHO meeting in Cape Town in 2006, it was suggested that developing countries will have to shift focus to alternative (i.e. cell-based) vaccine production platforms to meet vaccine demand [3]. Although these were not considered at the time, plant expression systems have significant advantages such as being safe, highly up-scalable and potentially cost-effective. The disadvantages include potentially complicated purification procedures and low recombinant protein yields [4].

A number of studies have focussed on the expression of various influenza antigens in plant systems, and specifically in tobacco plants (*Nicotiana* spp.). The influenza virus surface haemagglutinin (HA) glycoprotein, which elicits the primary neutralising immune response, is the main target for vaccine development [5]. Shoji and colleagues [6] (2008) transiently expressed HA from H3N2 (A/Wyoming/03/03) which was targeted to the endoplasmic reticulum (ER), in *Nicotiana benthamiana*[6]. The HA product yielded ~200 mg/kg fresh leaf weight (FW). Mett et al. [7] also transiently expressed the A/Wyoming/03/03 strain HA (stem domain and globular domain) and neuraminidase (NA) proteins fused to the enzyme lichenase (LicKM) in *N. benthamiana*. The antigens were also ER-targeted and yielded 100 mg HA/kg FW or 400 mg NA/kg FW. The antigens produced in both of the above studies elicited HA-specific immune responses in test animals.

Shoji et al. [8] expressed a truncated version of plant codon-optimised HA from H5N1 (A/Indonesia/05/05) in *N. benthamiana* plants. This HA protein lacked the transmembrane domain and native signal peptide, and accumulated in the ER at a level of approximately 60 mg/kg FW. Spitsin and colleagues [9] expressed H5N1 (A/Viet Nam/1203/2004) HA variants in plants, implementing both apoplast and ER targeting for both transient and stable transformation expression systems. The variants included a full-length HA (aa 1–549), a shortened version that contained the major antigenic domains (aa 1–330), a C-terminal truncated version (aa 1–277) as well as a version lacking the N-terminal region (aa 68–277). The 34 kDa C-terminal truncated variant accumulated to the highest levels, up to 1 mg HA/kg FW and 4 mg HA/kg FW for transient and stable transformation expression systems, respectively. D’Aoust and colleagues [10] expressed HA from the A/Indonesia/5/05 (H5N1) and the A/New Caledonia/20/99 (H1N1) strains by means of agroinfiltration in *N. benthamiana*. The HA was successfully expressed as virus-like particles (VLPs) budding from the plasma membranes of the plant cells, and accumulating between the plasma membrane and the cell wall. The yield of HA was about 50 mg/kg FW. All of the above studies confirmed that influenza HA can be expressed to high levels in plants, and that this plant-produced antigen can also induce HA-specific immune responses in animals.

As a proof of concept that plant-produced influenza vaccines are feasible for South Africa, from early 2006 we focussed on attempting to produce the HA of the highly pathogenic avian influenza virus (HPAIV) A/Viet Nam/1194/2004 (H5N1). To date, HPAIV H5N1 is the most virulent influenza strain, with a mortality rate of up to 60% in humans (WHO website, http://www.who.int/) and up to 100% in domestic birds [11]. Although human-to-human transmission is rare, the ability of H5N1 to infect humans combined with its tendency to mutate is still considered to be a serious global threat (http://www.who.int/). In this study, we optimised the transient expression of full length and truncated H5 HA variants in tobacco plants by means of agroinfiltration. We investigated targeting of the HA variants to different plant cell compartments: specifically, to the apoplastic spaces, the ER, the chloroplast as well as the cytosol. We also generated stable transgenic plant lines expressing these HA variants by focusing on the expression constructs that produced high transient HA protein concentrations. Immunogenicity trials with chickens and mice were conducted to determine whether the HA proteins were immunogenic, and haemagglutination and haemagglutination-inhibition (HI) assays to determine whether the proteins were correctly configured.

## Results

### Transient expression of H5 in tobacco plants

We compared the transient expression of eight H5N1 HA constructs (see Table 1) in *N. benthamiana* plants. These constructs successfully expressed both the full-length (H5) and truncated (H5tr) forms of HA in all of the subcellular compartments tested (ER, chloroplast, cytosol and apoplastic spaces)*.*

**Table 1:** Summary of the clones constructed during the project

**Clones**	**Cloning sites**	**Plant cell compartment targetted**
pTRAc-H5	*AflIII* and *XbaI*	Cytosol
pTRAc-H5tr		
pTRAERH-H5	*NcoI* and *NotI*	ER
pTRAERH-H5tr		
pTRACTP-H5	*MIuI* and *XbaI*	Chloroplast
pTRACTP-H5tr		
pTRAa-H5	*NcoI* and *XbaI*	Apoplastic spaces
pTRAa-H5tr		

The cloning sites as well as the targeted cell compartments are indicated.

**Figure 1:**
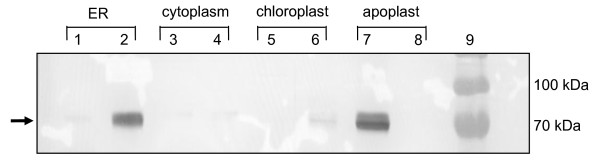
**Western blot analysis of H5 and H5tr transient expression in *N. benthamiana*, showing accumulation in various subcellular compartments 7 days post infiltration.** HA protein was detected using a primary rabbit anti-H5N1 polyclonal antibody. Crude plant extracts were analysed from plant tissue infiltrated with Agrobacterium strains carrying the following expression vectors: Lane 1, pTRAERH-H5; lane 2, pTRAERH-H5tr; lane 3, pTRAc-H5; lane 4, pTRAc-H5tr; lane 5, pTRACTP-H5; lane 6, pTRACTP-H5tr; lane 7, pTRAa-H5; lane 8, pTRAa-H5tr; lane 9, protein ladder (Fermentas). The arrow indicates the position of the H5 and H5tr proteins.

**Figure 2:**
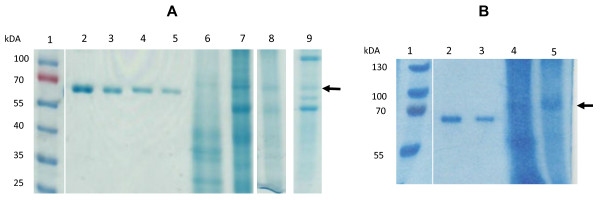
**A) Coomassie-stained SDS-PAGE gel of transiently expressed H5 and H5tr.** Crude, clarified extracts were prepared from *N. benthamiana* leaves that were expressing the H5 and H5tr proteins transiently. The extracts were separated by SDS-PAGE and compared to known BSA concentrations after staining with Coomassie Blue. Lane 1, prestained protein ladder (Fermentas); lane 2, 6.25 μg BSA; lane 3, 3.12 μg BSA; lane 4, 1.56 μg BSA; lane 5, 0.78 μg BSA; lane 6, concentrated H5, lane 7 concentrated H5tr, lane 8, crude H5 extract, lane 9 crude H5tr extract. The arrow indicates the position of the H5 protein. **B) Coomassie-stained SDS-PAGE gel of H5 expressed in stable transgenic plants.** Apoplast-targeted H5 from transgenic plants was separated lanes 4 and 5. Lane 1, protein ladder (Fermentas); lane 2, 5 μg BSA; lane 3, 2.5 μg BSA; lane 4, crude H5 extract; and lane 5, concentrated H5.

Expression time trials showed that optimum HA-expression levels occurred at approximately 7 days post infiltration (dpi). The engineered H5 and H5tr proteins were expressed as the uncleaved HA0 form, with products being approximately 70 kDa in size. The transient expression levels as determined by western blot for the various plant cell compartments are shown in Figure 1. It is clear from the western blot that the highest expression of full-length HA (H5) occurred in the apoplastic spaces (lane 7), while the highest accumulation of the truncated variant (H5tr) occurred in the ER (lane 2). These results were confirmed by three independent transient expression experiments. When protein production was scaled up by using the vacuum infiltration method on whole plants, we focussed on the two high-yielding constructs, namely pTRAa-H5 and pTRAER-H5tr. The H5 and H5tr proteins generated by this method were quantitated on Coomassie-stained gels after SDS-PAGE by comparison to known BSA concentrations (Figure 2A). Crude extracts of the H5 and H5tr proteins were separated in lanes 8 and lane 9, respectively. The expression levels obtained in these plant extracts ranged from 62–130 mg H5/kg fresh weight (FW) and 300–675 mg H5tr/kg FW.

In order to prepare plant-produced HA for animal trials, ground plant extracts were clarified and diafiltered to enrich and concentrate HA and to perform a buffer exchange. Diafiltration reduced the concentration of plant proteins, including RuBisCO (~ 55 kDa and 13 kDa subunits), while retaining most of the HA protein (approximately 67 % final yield). The final product still contained significant amounts of plant protein contaminants.

**Figure 3:**
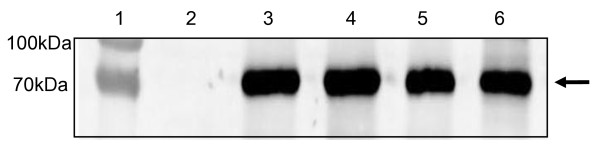
**Western blot analysis of HA products harvested from leaf apoplastic space extracts.***N. benthamiana* plants that were transiently transformed with pTRAa-H5 were infiltrated with buffer, following which the buffer was collected by low-speed centrifugation. HA was detected in the apoplastic space extracts using rabbit anti-H5N1 antibody. Lane 1, protein ladder (Fermentas); lane 2, wild type plant control; lanes 3 – 6, H5 products of different pTRAa-H5-infiltrated leaf batches.

Apoplast-accumulated HA protein was selectively extracted from *N. benthamiana* leaves 7dpi with pTRAa-H5. This was accomplished by infiltrating the leaves with buffer (PBS containing 0.1% Triton X-100) seven days after the agroinfiltration. The buffer was then collected from the cut edges of leaves by low speed centrifugation, effectively extracting H5 protein from the apoplastic spaces (Figure 3). Immobilized metal affinity chromatography was also successfully utilized to purify His-tagged H5tr protein (results not shown); however, yields were low and could not be reliably quantitated by SDS-PAGE analysis.

### H5 expression in transgenic tobacco plants

**Figure 4:**
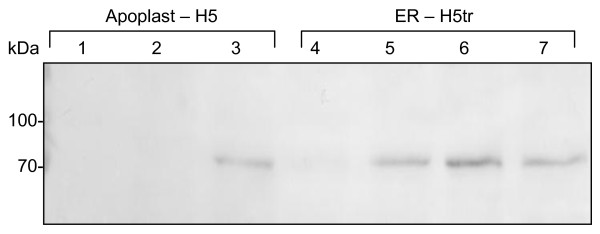
**Western blot analysis showing HA expression in a representative sample of transgenic plant lines.** Samples were harvested from T_3_ plant lines that were transformed with pTRAa-H5 (lanes 2–4) or pTRAERH-H5tr (lanes 5–7). Lane 1, protein ladder (Fermentas).

Stably transformed *N. tabacum* plants were generated with the pTRAa-H5 and pTRAERH-H5tr constructs. Three T_0_ pTRAa-H5 lines and ten pTRAERH-H5tr lines were allowed to self-pollinate, following which ~20 seeds from each line were planted. All H5 plant lines (T_1_) were PCR-positive indicating that all three plant lines probably have multiple HA gene inserts, only three of the ten H5tr plant lines were positive by PCR. These PCR positive plant lines were found to be positive for H5 or H5tr protein expression by western blot analysis. Gene transfer remained stable both in the second (T_2_) and third generations (T_3_) of H5 and H5tr transgenic plants, while these also successfully expressed H5 and H5tr, respectively (Figure 4). The H5tr accumulation in the stable T3 transgenic plants ranged from 160 – 880 mg/kg, which was lower than the levels obtained by transient expression. However, expression levels of H5 were higher in the stably transformed transgenic plants (640–1440 mg/kg) than in the transient expression system. The H5 protein from transgenic plants was concentrated and partially purified (Figure 2B, lane 5) as described for the transiently-expressed H5. The H5 enrichment is evident when the processed extract is compared to the crude plant extract (lane 4).

### Haemagglutination and haemagglutination-inhibition (HI) analyses

Haemagglutination and HI tests were conducted at the National Institute of Communicable Diseases (NICD, Sandringham, South Africa) a National Influenza Centre for WHO, in order to determine the functionality of the plant-produced HA protein variants. The crude pTRAa-H5 product gave a haemaglutination titre of 1:32 and a HI titre of 1:1024 against H5 antiserum (positive H5 in-house control gave 1:512). The truncated H5tr product gave a haemaglutination titre of 1:32; however, the HI titre was inconclusive. The HI tests were repeated to confirm the results.

**Table 2:** Summary of results obtained from chicken and mouse sera analysis

**Serum**	**H5-specific Western blot**	**HI test**
H5-immunised chickens (n = 4)	+	-
H5tr-immunised chickens (n = 4 )	+	+ (n = 2)
Control: PBS-immunised chickens (n = 2)	-	-
H5-immunised mice (n = 10)	+	+ (n = 3)
H5tr-immunised mice (n = 10 )	+	-
Control: PBS-immunised mice (n = 10)	-	-

### Animal serum analysis

Western blot analysis of the serum obtained from H5- and H5tr-immunised chickens showed that HA-specific antibodies were present in all the chicken sera: data are not shown due to the significant background signal observed due to chicken antibodies strongly binding the plant antigens present in the plant-produced HA preparations. Sera from the PBS-immunized control animals showed no reactivity to the HA protein.

From all the H5 and H5tr sera tested at the NICD, only two H5tr serum samples inhibited haemagglutination by control H5N1 virus during HI tests (titres of 1:32 and 1:8, respectively) (Table 2). The H5 ELISA and HI analysis performed by the Provincial Veterinary Laboratory were negative for H5-specific antibodies – however, the test was specific for an ostrich-derived low pathogenicity H5N2 virus, indicating a lack of cross-reactivity.

**Figure 5:**
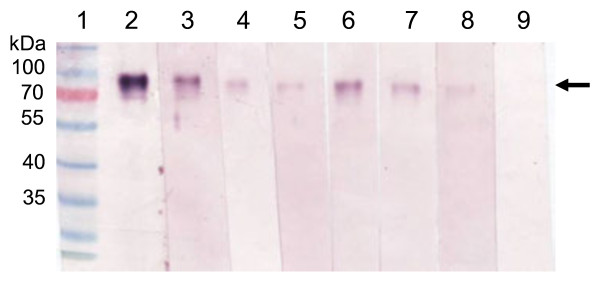
**Western blot indicating the presence of H5-specific antibodies in sera from mice immunised with plant-produced H5 or H5tr.** Purified H5N1 HA protein (Sino Biological) was detected using sera from H5-immunised mice (lanes 3–5), or H5tr-immunised mice (lanes 6–8). Lane 1, protein ladder (Fermentas); lane 2, HA detection using anti-H5N1 HA mouse monoclonal antibody (1:5000, Abcam) and lane 9 is the negative control where sera from animals vaccinated with PBS was used (dilution 1:4000). The arrow indicates the presence of the correct band size in all the H5 and H5tr candidate vaccine sera samples.

Sera from mice that were vaccinated with the plant-produced H5 or H5tr were analysed by western blot utilising a purified commercial H5 protein as antigen (Figure 5). This analysis indicated that H5-specific antibodies were present in the sera from all H5- (lanes 3–5) and H5tr- (lanes 6–8) immunised mice. H5 specific antibodies were absent in the control sera (from mice vaccinated with PBS lane 9). However, HI assays performed with the mouse sera were not conclusive, with only 3 of the mice vaccinated with full length H5 having antibody titres of 1:64 (Table 2).

## Discussion

Plant-produced vaccines could be the way of the future for pandemic influenza vaccine production, ever since two different groups showed that production time for a new H1N1 pandemic vaccine could be as short as one month from the first announcement of the pandemic strain sequence, to having the end-product ready for testing in animals [12]. This is significantly shorter than the 4 month minimum production time required for the seasonal vaccine made by egg-based technology. In addition, compared to plant-produced vaccines, egg-based vaccines also have the disadvantage that certain strains, such as H5N1, do not grow well in eggs and production is severely limited [3].

To illustrate the advantage for vaccine production via transient plant expression, when a deadly H3N2 influenza strain emerged in Brisbane, Australia (A/Brisbane/10/2007), researchers at the Fraunhofer Institute (USA) took 36 days to produce a purified candidate vaccine via transient expression in tobacco plants (800 mg/kg fresh plant material) [8]. In addition, a Canadian company (Medicago Inc.) were able to obtain purified H1-VLPs within 21 days after the initial pandemic H1N1 HA sequence became known [13]. Medicago and the Fraunhofer Institute (USA) also announced at the ‘Influenza Vaccine for the World 2009’ conference that human clinical trials will be implemented for their plant-produced HA influenza vaccines. These are currently in progress [14,15].

In our study, we aimed to establish a platform to produce influenza A virus subunit vaccines via tobacco plants in South Africa, in order to minimise the impact of a potential pandemic on our population. Pre-pandemic awareness and stockpiling has also been encouraged and is deemed necessary for H5N1 as it is still viewed as a potential global pandemic threat due to its high mortality rate [16]. In this case, stable transgenic seeds can be very useful to have on standby in case of a H5N1 pandemic. Our H5 and H5tr stable transgenic plant lines expressed vaccine protein at high levels for two consecutive generations – which is a first for transgenic production of influenza HA protein. When needed, these stored transgenic seeds can be planted at large scale, and the resulting transgenic plants can be used to purify H5 vaccines within a few weeks.

By targeting the HA variants to different plant cell compartments, we were able to determine which localities in the plant cell had the highest potential for the accumulation of our recombinant proteins. Our findings from both the transient and the stable transgenic systems indicated that full-length H5 accumulated at the highest level in the apoplastic spaces. This may be due to the presence of the HA transmembrane domain which can allow budding from the cell membrane in the form of VLPs, as observed by D’Aoust et al. [10]. In our study, the apoplast-targeted HA also provided us with a potential alternative to leaf homogenization which should reduce contaminating plant proteins and simplify the purification process. We were able to recover H5 protein from H5-expressing leaf tissue by infiltrating leaves with a buffer containing a detergent (Triton X-100), followed by low speed centrifugation of whole leaves. By using this method we were also able to extract H5 localised in the chloroplast or cytoplasm, but to a lesser extent (data not shown). This was likely due to the presence of Triton-X100 in the buffer, which allowed the elution of intracellular proteins by solubilising the membranes. This is an attractive alternative extraction method especially for apoplast-accumulating proteins, as it markedly reduces co-extraction of intracellular proteins and insoluble plant material.

H5tr accumulated at high levels in the ER. The ER is a popular target for expression of recombinant proteins, particularly glycoproteins, often resulting in high accumulation levels [17]. The contrasting behaviour of H5tr and H5 with respect to accumulation – the former high in the ER and low in the apoplast, with the inverse for H5 – is not easily explained. It is possible that SEKDEL-tagged H5 is less stable than the H5tr equivalent.

In comparison to other studies investigating HA expression in plants [6]‐[10] we obtained similar transient expression levels (62–675 mg/kg) than previously reported (1–800 mg/kg). Our choice of expression vector, infiltration methods and codon optimised HA gene sequence can possibly account for our high HA accumulation. Although immobilized metal affinity chromatography (IMAC) purification (via His-tag) has previously been successful for this protein [8], we could not utilize the same purification strategy for our full-length H5, as it did not contain a His-tag. We were able to purify the His-tagged truncated H5 by IMAC; however, our yields were too variable and too low for us to use the purified material for animal experiments. We therefore decided to generate our preliminary data using a crude concentrated and diafiltered product, in which the HA proteins were enriched. More important, though, is not the amount of protein produced, but the fact that the plant-produced H5 protein exhibited the correct conformation as shown by the success of HA and HI tests in comparisons with known standards.

The murine trial demonstrated the ability of plant-produced H5 and H5tr to induce HA-specific antibodies, which was verified by western blot. The candidate H5 and H5tr vaccines therefore elicited the correct antibody response *in vivo*. However the HI titres were relatively low for all the sera. Results for the sera of mice vaccinated with H5 showed that only three had HI titres above 1:64 the minimum HI titre considered to be protective in humans is ≥ 1:40 [18] which indicates that the plant-produced H5 in this study would have elicited a protective immunity. The HI titres for mice vaccinated with H5tr were inconclusive. One of the reasons that the full length H5 did not elicit high HI titres might be due to the extraction method employed. The addition of Triton-X100 to the extraction buffer possibly also destroyed any VLPs that were formed.

Western blot analysis of H5-immunised chicken sera clearly demonstrated the presence of HA-specific antibodies. HA-specific antibodies were also present in the sera from H5tr-immunised chickens, but to a lesser degree. In general, the antibody concentration was too low to inhibit haemagglutination of red blood cells during HI testing, with the exception of two serum samples from the H5tr-immmunised chickens (1:16 and 1:32). Even though the HI titres were lower than 1:40 in both mice and chickens immunised with H5tr, it still indicated that our candidate H5tr vaccine warrants further investigation.

HI titres were relatively low for all chicken and mice sera: immune responses to our vaccine candidates may have been suboptimal because of dose and adjuvant choice (IFA). Other studies that have tested plant-produced HA subunit vaccine candidates in animals have used various influenza strains, regions of HA, purification protocols, dosages, adjuvants and test animals - all which may play a role in vaccine efficacy. In mice, Shoji *et al*. [6] tested three doses ranging from 5 μg to 30 μg together with 10 μg of Quil A adjuvant. D'Aoust [10] administered two doses of 0.5 μg H5-VLPs and Spitsin *et al.*[9] used two doses of 10 μg with alum-CpG adjuvant. When considering immunisation studies conducted on ferrets, the doses varied from a 100 μg with alum adjuvant [7] to 45 and 95 μg HA in Quil A adjuvant [8]. Ferrets, which are the preferred animal model for influenza [19], were not available for our study; we decided to conduct our vaccine trial on chickens since these can be naturally infected by H5N1. We deduced that our vaccine regimen (three doses of 13 μg antigen) may have been too low to induce high antibody titres in chickens. A dose-ranging study will be necessary to determine the optimum antigen and adjuvant dosage. As mentioned above, the full-length H5 antigen may not have elicited high HI titres due to the presence of Triton-X100 in the extraction buffer, which may have destroyed any VLPs that were formed. D’Aoust [10] showed that low doses of 0.5 μg VLPs protect against lethal challenge in mice and that H5 antigen which did not form VLPs induced up to six fold lower HI responses. It would be interesting to determine if VLPs were formed in our study and to develop extraction methods for them.

Based on our current results, it is not clear whether the plant-produced H5 or the H5tr showed the best potential as a candidate subunit vaccine. Overall, a higher concentration of H5-specific antibodies was detected in chicken than in mouse sera; however, only two of the H5tr chicken serum samples and three of the H5 mouse serum samples gave low positive HI results.

## Conclusions

Our findings indicate it is possible to produce influenza subunit vaccines via plants in South Africa. The yields of H5 and H5tr varied with experiment and by transgenic plant line, but in general were higher than those obtained by others: this may be due to the vector system used, as well as to the human codon optimisation of the genes. This is novel for HA genes in plants, and is based on our previous observation of a significant yield improvement for HPV L1 proteins [20]. These yields should make it economical to produce the H5 vaccine, even given the fact that higher doses of novel influenza vaccines will probably be needed in comparison to the seasonal vaccines [14]. We still need to give more attention to downstream processing and especially purification techniques, as large-scale end-stage production facilities do not exist in South Africa. The study remains ongoing, as we are still monitoring our stable transgenic plants to determine whether gene integration remains stable over the next few generations: this will determine whether or not we will be able to establish a stable seedbank for emergency large-scale vaccine production.

## Methods

### Strain selection and synthesis

After screening numerous AIV HA genes, the complete HA gene (H5; 1 704 bp) of the A/Viet Nam/1194/2004 strain (H5N1, GenBank accession number: AY651333) was selected to be the consensus amino acid sequence for developing a candidate subunit vaccine. In addition, a 23 amino acid-truncated form of the H5 gene (H5tr; 1 635 bp) was also engineered by removing the membrane-anchoring domain coding region (nucleotides 1 597 – 1 665 bp). The removal of the membrane-anchoring domain was intended to prevent membrane insertion of the protein, making it soluble and therefore aiding in purification. Both HA variants (H5 and H5tr) were human-codon optimised and synthesised (GENEART, Germany). Specific restriction enzyme sites were added during gene synthesis to facilitate cloning.

### Construction of plant expression vectors

The H5 and H5tr genes were cloned into binary plant expression pTRA vectors, described previously [see [20,21] for details]. These vectors are able to direct protein expression to different plant cell compartments; namely, the cytoplasm (pTRAc), chloroplast (pTRAkc-rbcs1-cTP), ER (pTRAkc-ERH) and the apoplastic spaces (pTRAkc-A). The pTRAkc-ERH vector added a 6 X histidine (His) C terminal tag to facilitate purification. The apoplastic space-targeted clones were created by cloning the HA genes into the pTRAkc-ERH vector following removal of the His-tag and ER retention signal (SEKDEL) sequences. See Table 1 for cloning details and resulting clones.

### *Agrobacterium tumefaciens-*mediated transient expression

The HA expression constructs were transformed into *A. tumefaciens* GV3101(pM90RK) cells by electroporation as described [20]. Recombinant *Agrobacterium* was grown in LB media supplemented with 50 μg carbenicillin/ml, 30 μg kanamycin/ml and 25 μg rifampicin/ml to maintain selection of transformants.

*Agrobacterium* cultures containing the HA expression constructs were cultured in induction medium (LB broth, 10 mM MES, 20 μM acetosyringone and 2 mM MgSO4, pH 5.6) containing antibiotics (50 μg carbenicillin/ml, 30 μg kanamycin/ml and 25 μg rifampicin/ml) at 27°C overnight. In addition, the *Agrobacterium* LBA4404 (pBIN-NSs) strain (obtained from Marcel Prince, Laboratory of Virology, The Netherlands) was also cultured. This strain contains a silencing suppressor gene of tomato spotted wilt virus (TSWV) to prolong and increase recombinant gene expression [22]. *Agrobacterium* was cultured to exponential phase (OD600 = 0.8) and centrifuged at low speed (4000 x g). The pellet was resuspended in infiltration buffer (10 mM MES, 10 mM MgCl_2_, 150 μg acetosyringone/ml and 2% sucrose) and left at room temperature for three hours. Each HA-containing *Agrobacterium* strain was combined with LBA4404 (pBIN-NSs) and diluted with infiltration buffer to generate a final OD600 of 0.25 for each strain (total *Agrobacterium* OD600 of the suspension was 0.5).

A time trial was conducted to determine the day with the highest HA expression level. The *Agrobacterium* cultures were co-infiltrated directly into the abaxial air spaces of *N. benthamiana* leaves using a syringe. Infiltrated *N. benthamiana* plants were grown at 22°C with 16 h light and 8 h dark cycles, with light intensity of 60–80 μE/m^2^/s. Five leaf disks (~ 8 mm diameter, ~ 13 μg) were harvested from each plant at intervals for 8 days. The leaf disks were ground in liquid nitrogen, followed by the addition of 500 μl of Tris–HCl, pH8.

Larger scale expression studies were performed by vacuum infiltration of whole *N. benthamiana* plants (up to 20 plants) [20]. Vacuum infiltrated plants were harvested and processed seven days post-infiltration. The infiltrated leaves were ground in liquid nitrogen and suspended in 100 mM Tris–HCl pH 8 (1:2 ratio; mass: volume). HA proteins that had accumulated in leaf apoplastic spaces were recovered by vacuum or syringe infiltrating buffer (PBS containing 0.1% Triton X-100) into the leaves, following which the leaves were cut into strips about 2 cm wide, layered on plastic grids and centrifuged at low speed to liberate the protein eluate.

### *Nicotiana tabacum* stable transformation and regeneration

*N. tabacum* L. ‘Petite Havana’ SR1 plants were cultivated axenically on Murashige and Skoog (MS) media at 22°C with 16 h-light and 8 h-dark photoperiod, at light intensity of 60–80 μE/m^2^/s. *N. tabacum* leaf discs were transformed by binary expression vectors in *A. tumefaciens* using standard techniques [20,23]. Based on high transient expression results, the ER-targeted constructs (pTRAERH-H5 and pTRAERH-H5tr) and apoplast-targeted constructs (pTRAa-H5 and pTRAa-H5tr) were used for stable transformation. Rooted primary (T_0_) transformants were transferred into soil, flowering plants were self-pollinated and T_1_ seeds were collected. Primary T_1_ progeny were selected on MS medium supplemented with 120 μg/ml kanamycin. The plants were propagated into T_2_ populations.

DNA was extracted from the first generation (T_1_) transformed *N. tabacum* plants using the CTAB method [24]. The DNA was screened by PCR for the presence of the HA genes using internal gene primers, namely H5SeqFw_633 5’ GACCAAGCTGTACCAGAACC 3’ and H5SeqRev_1132 5’ GCTCATTGCTGTGGTGGTA 3’, which produced a 518 bp fragment.

Twenty five plants were analysed for protein expression by western blots. Protein was extracted randomly from five leaf disks. The plants that were positive for the HA expression were self-fertilized to generate a homozygous second generation and third generation plants. Each generation was screened by PCR and followed by western blot analysis.

### Protein analysis and purification

For both transient and stable transgenic expression systems, the HA accumulation was evaluated by means of western blotting. Crude plant extract was mixed with SDS sample buffer, heated, and separated on 10% SDS-PAGE gels. The gels were transferred to nitrocellulose membranes by means of semi-dry electroblotting (Bio-Rad). HA protein was detected using a primary rabbit anti-H5N1 polyclonal antibody (1:500 dilution, GenBank strain reference: AAT76166, US Biological) in conjunction with a secondary goat anti-rabbit antibody (1:7000, Sigma). Nitro blue tetrazolium chlode/5-bromo-4 chloro-3-indolyl phosphate (NBT/BCIP) tablets (Roche) were used as the detection substrate. To quantify the amount of antigen produced, we compared the band intensities of HA and known BSA concentrations on Coomassie-stained SDS-PAGE gels by densitometry (Gene Genius Bioediting system, Syngene).

The apoplast-targeted full-length H5 from T_2_ transgenic *N. tabacum* plants or from vacuum infiltrated *N. benthamiana* plants was extracted using Dubecco’s PBS (Sigma) containing 0.1% Triton –X100. The ER-retained truncated H5 (H5tr) was extracted from vacuum infiltrated *N. benthamiana* plants using 100 mM Tris–HCl, pH8. Generally, 20 g of leaf material was ground up using a mortar and pestle in liquid nitrogen and suspended in 40 ml extraction buffer (1:2 ratio; mass: volume). Crude plant extracts were filtered through Miracloth and clarified by centrifugation. HA proteins were enriched and concentrated by diafiltration into Dulbecco’s PBS (Sigma) using ultrafiltration hollow fiber cartridges (50 kDa MWCO, Amersham Biosciences). This resulted in buffer exchange of Tris–HCl to PBS for H5tr and the removal of Triton-X100 from H5. These HA proteins were subsequently used in the animal trials. In addition, to capture the His-tagged H5tr proteins, the crude extract of the ER-targeted H5tr protein was filtered through Miracloth and then affinity purified using Ni-NTA resin (Qiagen).

### Haemagglutination and Haemagglutination inhibition assays

Purified plant-produced HA protein samples (H5 and H5tr) were freeze-dried and sent to the National Institute for Communicable Diseases (NICD) (Johannesburg, South Africa) for haemagglutination and haemagglutination inhibition (HI) tests to determine their functionality. Serial dilutions of known H5 antisera were tested against our plant-produced protein or a standard dose (4HAU/25 μl) of Influenza A (H5N1) control antigen (obtained from CDC, Atlanta). After confirming the functionality of the plant-produced HA proteins, animals (chickens and mice) were vaccinated with these plant-produced proteins and their sera were tested in a HI assay against Influenza A (H5N1) control antigen (4HAU/25 μl). Following incubation, a 0.5 % suspension of Turkey red blood cells (RBC) was added and the test interpreted by the inhibition of haemagglutination of RBC. Influenza A (H3N2) and Influenza A (H1N1) antigens were used as negative controls to determine whether HI cross reaction was observed. Inactivated antigens were obtained from Center for Disease Control (CDC) in Atlanta, USA.

### Animal trials and serum analysis

Ten Rhode Island Red male chickens (< 12 weeks old; Provincial Veterinary Laboratory, Stellenbosch, South Africa) were pre-bled three days prior to vaccination. The chickens were divided into three groups with the candidate vaccine groups (group 1 and 2) comprising of four chickens each while the control group (group 3) comprised of two chickens. Group 1 and 2 received a dose of transiently expressed 13 μg plant-produced protein suspended in 1 ml Dubecco’s PBS mixed with incomplete Freund’s adjuvant (IFA), at a 1:1 ratio. Group 1 received H5 and group 2 H5tr while group 3 received Dulbecco’s PBS. Administration of the vaccines occurred on day 0, 14, 28 and the chickens were sacrificed according to the animal ethical code stipulated by UCT on day 42. Two weeks after each vaccination, a 1 ml blood sample was collected from each chicken. In addition to the HI assays conducted at the NICD as described above, the chicken sera were screened by HI assays and ELISA using antibodies specific to a low pathogenicity H5N2 virus isolated from ostriches in South Africa.

In the mouse immunisation trial, thirty 7 week old Balb/c mice were pre-bled three days prior to vaccination. The mice were separated into three groups of 10 mice per group. Group 1 and 2 were immunised with 8 μg (100 μl) plant-produced H5 from transgenic plants and transiently expressed H5tr, respectively, each mixed with IFA (1:1). Group 3 or the control group received Dulbecco’s PBS. The candidate vaccine was administrated intramuscularly to mice, 50 μl into each anterior *tibialis* muscle. Four doses were administrated at two weeks intervals (day 0, 14, 28 and 31) and blood was collected before each dose, and 11 days after the final dose when the animals were euthanized (day 42). The sera were stored at −20°C for further analysis. The sera were transported to the NICD for HI assays as described previously. All animal experiments were approved by the UCT’s Animal Ethics committee (HSFAEC009/001).

To determine the presence of HA-specific IgG antibodies, all the sera were screened by western blot. Plant-produced H5 and H5tr protein was separated on an SDS-PAGE gel and blotted onto nitrocellulose membrane. Following protein transfer, the membrane was cut into strips and probed with chicken sera (1:1000 dilutions). After the washing steps, rabbit anti-chicken IgY (IgG) antibody (Sigma) was used to determine whether H5-specific antibodies were present. For the mouse sera, human cell-expressed and purified H5 protein from (A/VietNam/1194/2004) H5N1 (Sino Biological Inc) with 99% similarity to the plant expressed H5 was loaded onto a gel and membrane strips were probed with 1:4000 sera dilutions, and goat anti-mouse IgG (Sigma) secondary antibody.

## Competing interest

The authors declare that they have no competing interest.

## Authors' contributions

EM carried out transient expression experiments, carried out animal experiments and drafted the manuscript, JMM created the expression constructs and the transgenic plants, participated in design of the study and helped to draft the manuscript, SM selected transgenic plants, purified the proteins, participated in the mouse experiments and did serum analysis, AB did the HI essays on plant product and serum of vaccinated animals, ALW conceived the study and wrote original project proposal, IIH designed and coordinated the study, helped drafting the manuscript and revised the paper, EPR conceived the study, made the H5N1 strain selection, participated in design of study and drafting of manuscript. All authors read and approved of the final manuscript.
